# Suppression of Osteopontin Functions by Levocetirizine, a Histamine H_1_ Receptor Antagonist, *In Vitro*

**DOI:** 10.1155/2013/735835

**Published:** 2013-12-30

**Authors:** Toshimitsu Komatsuzaki, Isao Suzaki, Kojiro Hirano, Ken-Ichi Kanai, Kazuhito Asano, Harumi Suzaki

**Affiliations:** ^1^Department of Otorhinolaryngology, School of Medicine, Showa University, 1-5-8 Hatanodai, Shinagawa-ku, Tokyo 142-8555, Japan; ^2^Division of Physiology, School of Nursing and Rehabilitation Sciences, Showa University, Yokohama 226-8555, Japan

## Abstract

*Objectives*. Osteopontin (OPN), a multifunctional glycoprotein secreted from a wide variety of cells after inflammatory stimulation, is well accepted to contribute to the development of allergic diseases. However, the influence of histamine H_1_ receptor antagonists (antihistamines) on OPN functions is not well understood. The present study was undertaken to examine the influence of antihistamines on OPN functions *in vitro*. *Methods*. Human nasal epithelial cells (5 × 10^5^ cells) were stimulated with 250 ng/mL OPN in the presence of either desloratadine (DL), fexofenadine (FEX), or levocetirizine (LCT). The levels of OPN, GM-CSF, Eotaxin, and RANTES in 24 h culture supernatants were examined by ELISA. The influence of LCT on mRNA expression and transcription factor activation in cells were also examined by real-time RT-PCR and ELISA, respectively. *Key Findings*. The antihistamines examined significantly suppressed the production of GM-CSF, Eotaxin, and RANTES from cells after OPN stimulation. LCT also exhibited the suppression of mRNA expression for chemokines and transcription factor, NF-**κ**B and AP-1, activation, which were increased by the stimulation of cells with OPN. *Conclusions*. The suppressive activity of LCT on OPN functions on nasal epithelial cells may be responsible for the attenuating effect of the agent on allergic diseases.

## 1. Introduction

Osteopontin (OPN), also known as early T lymphocyte activation 1 (Eta-1), is a secreted multifunctional glycoprotein, which is produced by a large variety of cells, including macrophages, activated T cells, dendritic cells, and epithelial cells after inflammatory stimulations [1]. As the name implies, OPN stimulates the adhesion of osteoclasts to bone, and bone resorption is blocked by this interaction [2, 3]. OPN may also contribute directly to the regulation of mineral crystal formation [3]. In addition to these effects of OPN on bone metabolism, many studies have demonstrated that OPN is responsible for the development of Th1 T cell-mediated immune diseases, such as rheumatoid arthritis [4], tuberculosis [5], and multiple sclerosis [6]. On the other hand, the involvement of OPN in the Th2-associated allergic responses was extensively investigated and revealed that OPN may be implicated in the development of lower airway allergic inflammatory diseases, such as asthma [7, 8]. It is also observed that OPN could augment both IgE-mediated mast cell degranulation and mast cell chemotaxis *in vitro* [9]. Furthermore, OPN is reported to be able to suppress antigen-specific production of IL-13, which is responsible for the enhancement of both proinflammatory cytokine production from macrophages and IgE production, when CD4^+^ T cells were treated with OPN *in vitro* [10].

Allergic rhinitis (AR) is an allergic inflammation in the nasal mucosa. It occurs when an allergen, such as pollen, dust, or animal dander is inhaled by an individual with a sensitized immune system [11]. In such individuals, the allergen causes the production of IgE, which binds to receptors on the surface of mast cells and basophils. On reexposure to the relevant allergens, cross-linking of adjacent IgE molecules occurs, and the degranulation of mast cells and basophils takes place, releasing a wide variety of chemical mediators, including histamine and leukotriene, which cause sneezing, itching, runny nose, and congestion, among others [11]. From these established concepts, the best treatment is to avoid (or minimize) what causes allergic symptoms. Since it may be impossible to completely avoid all triggers, the medications using histamine H_1_ receptor antagonists (antihistamines), decongestants, and corticosteroids are recommended to relieve some allergic symptoms [12]. Although the administration of corticosteroids in mice could inhibit OPN production, which was increased by antigenic stimulation [13], the influence of antihistamines on OPN functions is not clear at present.

Cetirizine (CT) is one of the most potent second-generation antihistamines and is used for the treatment of allergic diseases, such as allergic rhinitis and urticaria [14]. Recently, levocetirizine (LCT), a third-generation nonsedative antihistamine, developed from the second-generation antihistamine CT and like CT it is a long lasting antihistamine covering the same area of allergic disorders [14, 15]. Chemically, LCT is the active enantiomer, *R*-enantiomer, of CT. Several studies suggest that LCT has a twofold increased affinity for histamine H_1_ receptors over that of CT and possesses higher receptor occupancy at 24 h than other antihistamines, including fexofenadine [16]. However, the influence of CT and LCT on OPN functions is not clear at present. The present study, therefore, was undertaken to examine the influence of CT and LCT on OPN functions using an *in vitro* cell culture technique.

## 2. Materials and Methods

### 2.1. Agent

CT, LCT, and desloratadine (DL) were purchased from Toronto Research Chem., Inc. (North York, ON, Canada) as preservative free pure powders. Fexofenadine hydrochloride (FEX) was kindly donated from SANOFI Co., Ltd. (Paris, France). They were dissolved in SABM medium (Lonza Co., Ltd., Walkersville, MD, USA) at appropriate concentrations for the experiments just before use.

### 2.2. Cell Source and Epithelial Cell Culture

Nasal polyp specimens were surgically obtained from chronic sinusitis patients who had not received any medical treatment, including systemic and topical steroid application or oral histamine H_1_ receptor antagonists underwritten informed consent, which was approved by the Ethics Committee of Showa University. Specimens were washed 5 times with PBS that contained 500 *μ*g/mL streptomycin, 500 U penicillin, and 5 *μ*g/mL amphotericin B. These tissues were then treated with 0.1% protease type XIV for 12 h at 4°C. Epithelial cell layers were then obtained and vigorously mixed with a pipet to obtain single cell suspension. The cells were suspended in SABM medium (Lonza Co., Ltd.) at a concentration of 5 × 10^3^ cells/mL. The cell suspension was introduced into 24-well tissue culture plates in triplicate that were coated with human Type I collagen and cultured for 48 h, when the number of cells reached approximately 5 × 10^5^ cells/well. Epithelial cells were then stimulated with either recombinant human TNF-*α* (R & D Systems, Inc., Minneapolis, NM, USA) or recombinant human OPN (R & D Systems Inc.) in the presence of various concentrations of antihistamines, such as CT, LCT, DL, and FEX. After 24 h, culture supernatants were obtained and stored at −80°C until used. In cases of examining mRNA expression and transcription factor activation, epithelial cells were cultured in a similar manner for 12 h and 4 h, respectively, and the cells were stored at −80°C until used.

### 2.3. Assay for factors

Factor levels, OPN, GM-CSF, Eotaxin, and RANTES in 24 h culture supernatants were examined by commercially available ELISA test kits (R & D Systems Inc.). The minimum detectable levels using ELISA test kits were 0.011 ng/mL for OPN, 3.0 pg/mL for GM-CSF, 2.0 pg/mL for RANTES, and 5.0 pg/mL for Eotaxin.

### 2.4. Assay for mRNA Expression

Poly A^+^ mRNA was separated from cultured cells with oligo (dT)-coated magnetic microbeads (Milteny Biotec, Bergisch Gladbach, Germany). The first-strand cDNA was synthesized from 1.0 *μ*g of Poly A^+^ mRNA using a Superscript cDNA synthesis kit (Invitrogen Corp., Carlsbad, CA, USA) according to the manufacturer's instructions. Polymerase chain reaction (PCR) was then carried out using a GeneAmp 5700 Sequence Detection System (Applied Biosystems, Forster City, CA, USA). The PCR mixture consisted of 2.0 *μ*L of sample cDNA solution (100 ng/*μ*L), 25.0 *μ*L of SYBR-Green Mastermix (Applied Biosystems), 0.3 *μ*L of both sense and antisense primers, and distilled water to give a final volume of 50.0 *μ*L. The reaction was conducted as follows: 4 min at 94°C, followed by 40 cycles of 15 s at 95°C and 60 s at 60°C. GAPDH was amplified as an internal control. mRNA levels were calculated by using the comparative parameter threshold cycle and normalized to GAPDH. The primer sequence used for real-time RT-PCR was as follows [12, 17, 18]: for GM-CSF, 5′-CGG AGT ACT GTA GCC ACA TGA TTG G-3′ (sense) and GAT TGG CGG TGT TAT TCT CTG AAG CG-3′ (antisense), for Eotaxin, 5′-AAG GCC CCT CAT TCA TCA G-3′ (sense) and 5′-TTC CTT GGA AAA TGC CTT TG-3′ (antisense), for RANTES, 5′-ACC ATG AAG GTC TCC GCG-3′ (sense) and 5′-TTC AGG TTC AAG GCA TCT CCA-3′ (antisense), and for GAPDH, 5′-ATC TGG CAC CAC ACC ACA TTC TAC AAT GAG CTG CG-3′ (sense) and 5′-CCG CAT ACT CCT GCT TGC TGA TCC ACA TCT GC-3′.

### 2.5. Assay for Activation of Both NF-*κ*B and AP-1

NF-*κ*B activity in cultured-epithelial cells was analyzed by commercially available NF-*κ*B ELISA test kits (Active Motif Co. Ltd., Carlsbad, Calf, USA) that contained sufficient reagents and monoclonal antibody against p65, according to the manufacturer's recommendation. In brief, nuclear extract (5.0 mg of protein) from cultured cells was introduced into each well of 96-well plates coated with oligonucleotide containing the NF-*κ*B consensus site (5′-GGGACTTTCC-3′) in a volume of 20.0 *μ*L, followed by incubation for 1 h at 25°C. After washing 3 times, 100 *μ*L monoclonal antibody against p65 was added to the appropriate wells and incubated for a further 1 h at 25°C. Anti-IgG HRP conjugate in a volume of 100 *μ*L was then added and further incubated for 1 h at 25°C. The absorbance at 450 nm was measured after the addition of TMB solution. AP-1 activity was also measured with commercially available ELISA test kits (Active Motif Co.) in a similar manner.

### 2.6. Statistical Analysis

All data are expressed as means ± SE. All results were analyzed using one-way analysis of variance. The level of statistical significance was set at *P* < 0.05.

## 3. Results

### 3.1. Influence of Antihistamines on OPN Production from Epithelial Cells after TNF-*α* Stimulation

**Figure 1:**
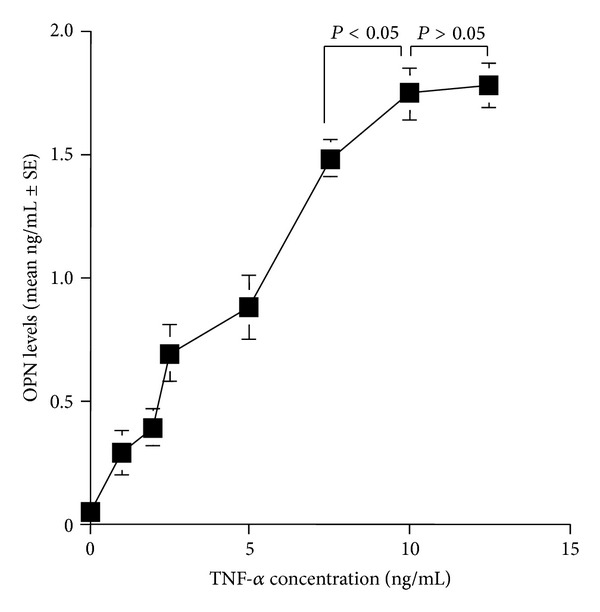
Influence of TNF-*α* on the production of osteopontin (OPN) from nasal epithelial cells *in vitro*. Nasal epithelial cells from five different subjects were stimulated with different concentrations of TNF-*α*. Culture supernatants were obtained 24 h later, and OPN levels were examined by ELISA.

**Figure 2:**
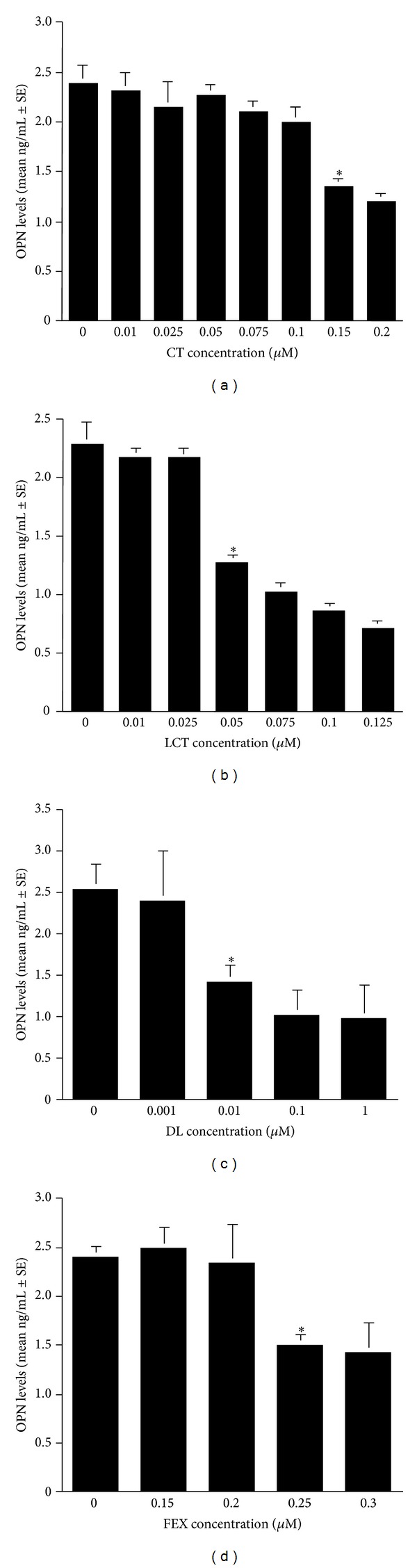
Influence of antihistamines on TNF-*α*-induced osteopontin (OPN) production from nasal epithelial cells *in vitro*. Nasal epithelial cells obtained from five different subjects were stimulated with 10 ng/mL TNF-*α* in the presence of various concentrations of CT, LCT, DL and FEX for 24 h. OPN levels in culture supernatants were examined by ELISA. *Significant (*P* < 0.05) versus control; (a) cetirizine (CT); (b) levocetirizine (LCT); (c) desloratadine (DL); (d) fexofenadine (FEX).

The first experiments were designed to examine whether human nasal epithelial cells could produce OPN in response to TNF-*α* stimulation and the optimal concentration of TNF-*α* to produce OPN. To do this, epithelial cells were stimulated with various concentrations (1.0 ng/mL to 12.5 ng/mL) of TNF-*α* for 24 h. OPN levels in culture supernatants were examined by ELISA. As the concentration of TNF-*α* used for stimulation was increased, the level of OPN in culture supernatants also increased (Figure 1). The maximum production was observed when the cells were stimulated with TNF-*α* at more than 10.0 ng/mL (Figure 1). We then examined the influence of antihistamines, CT, LCT, DL, and FEX, on OPN production from nasal epithelial cells in response to TNF-*α* stimulation. Epithelial cells were stimulated with 10.0 ng/mL TNF-*α* in the presence of either CT (0.01 *μ*M to 0.2 *μ*M), LCT (0.01 *μ*M to 0.125 *μ*M), DL (0.001 *μ*M to 1.0 *μ*M), or FEX (0.15 *μ*M to 3.0 *μ*M). As shown in Figure 2(a), treatment of cells with CT at less than 0.1 *μ*M scarcely affected OPN production after TNF-*α* stimulation: the levels of OPN in experimental culture supernatants contained similar levels of OPN to that observed in control. On the other hand, CT at more than 0.15 *μ*M caused significant suppression of OPN production induced by TNF-*α* stimulation (Figure 2(a)). We then examined the influence of LCT on OPN production from epithelial cells after TNF-*α* stimulation. As shown in Figure 2(b), culture supernatants obtained from cells treated with LCT at less than 0.025 *μ*M contained similar levels of OPN to that observed in control supernatants. However, when cells were treated with LCT at more than 0.05 *μ*M, OPN levels in culture supernatants significantly decreased as compared to that of control (Figure 2(b)). We finally examined the influence of DL and FEX on OPN production from nasal epithelial cells in response to TNF-*α* stimulation. As shown in Figures 2(c) and 2(d), these two agents also inhibited OPN production from cells after TNF-*α* stimulation. The minimum concentration that caused significant inhibition was 0.01 *μ*M for DL and 0.25 *μ*M for FEX, respectively.

### 3.2. Influence of Antihistamines on Factor Production from Epithelial Cells

**Figure 3:**
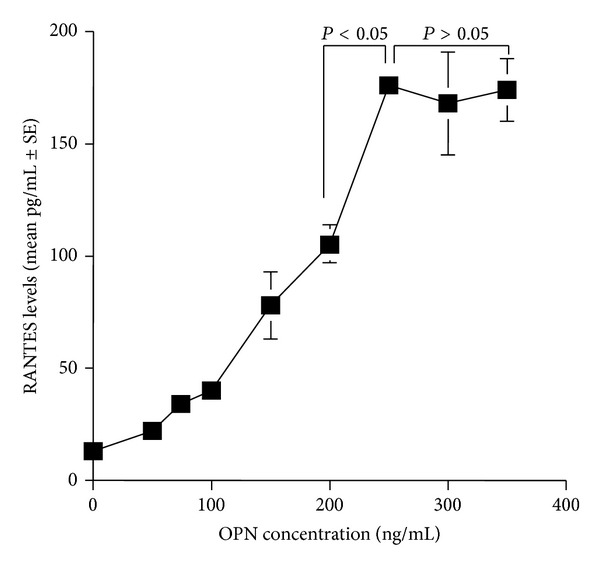
Influence of osteopontin (OPN) on RANTES production from nasal epithelial cells *in vitro*. Nasal epithelial cells obtained from five different subjects were stimulated with various concentrations of OPN for 24 h. RANTES levels in culture supernatants were examined by ELISA.

**Figure 4:**
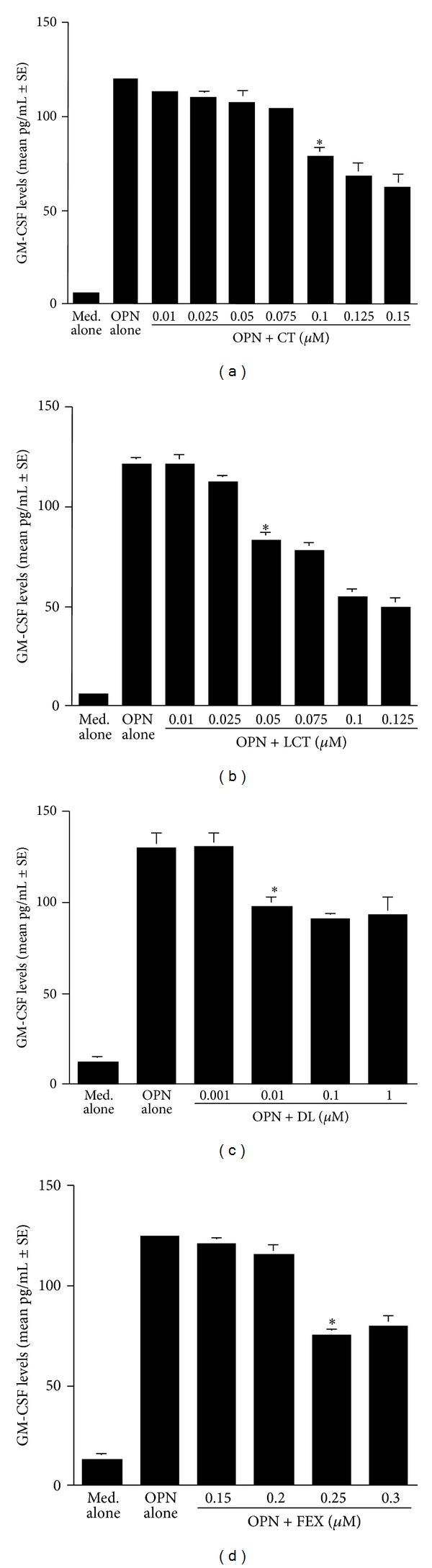
Influence of antihistamines on osteopontin (OPN)-induced GM-CSF production from nasal epithelial cells *in vitro*. Nasal epithelial cells obtained from five different subjects were stimulated with 250 ng/mL OPN in the presence of various concentrations of CT, LCT, DL and FEX for 24 h. GM-CSF levels in culture supernatants were examined by ELISA. *Significant (*P* < 0.05) versus OPN alone; (a) cetirizine (CT); (b) levocetirizine (LCT); (c) desloratadine (DL); (d) fexofenadine (FEX).

**Figure 5:**
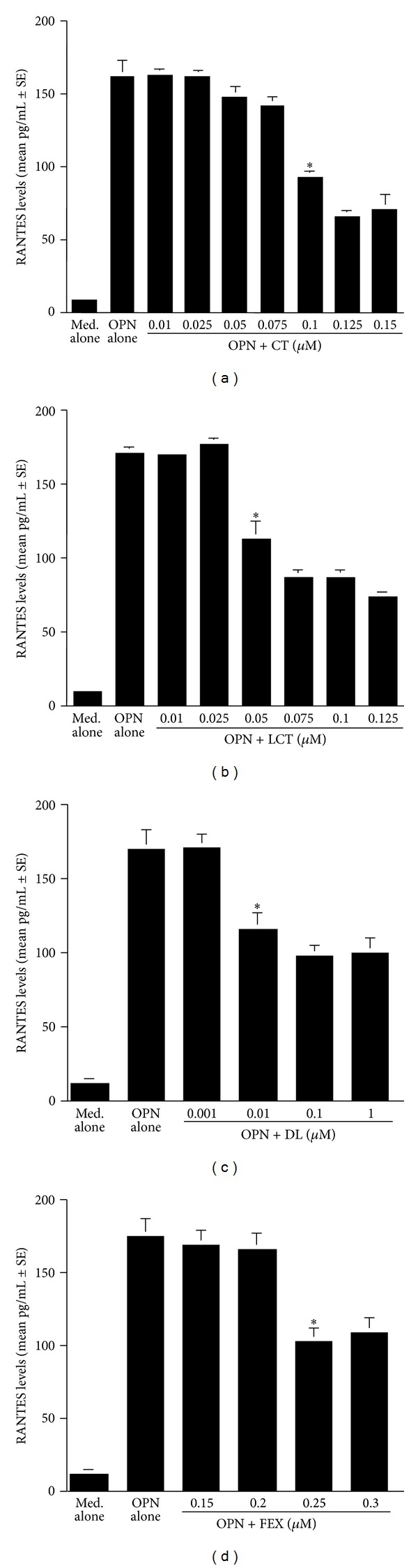
Influence of antihistamines on osteopontin (OPN)-induced RANTES production from nasal epithelial cells *in vitro*. Nasal epithelial cells obtained from five different subjects were stimulated with 250 ng/mL OPN in the presence of various concentrations of CT, LCT, DL and FEX for 24 h. RANTES levels in culture supernatants were examined by ELISA. *Significant (*P* < 0.05) versus OPN alone; (a) cetirizine (CT); (b) levocetirizine (LCT); (c) desloratadine (DL); (d) fexofenadine (FEX).

**Figure 6:**
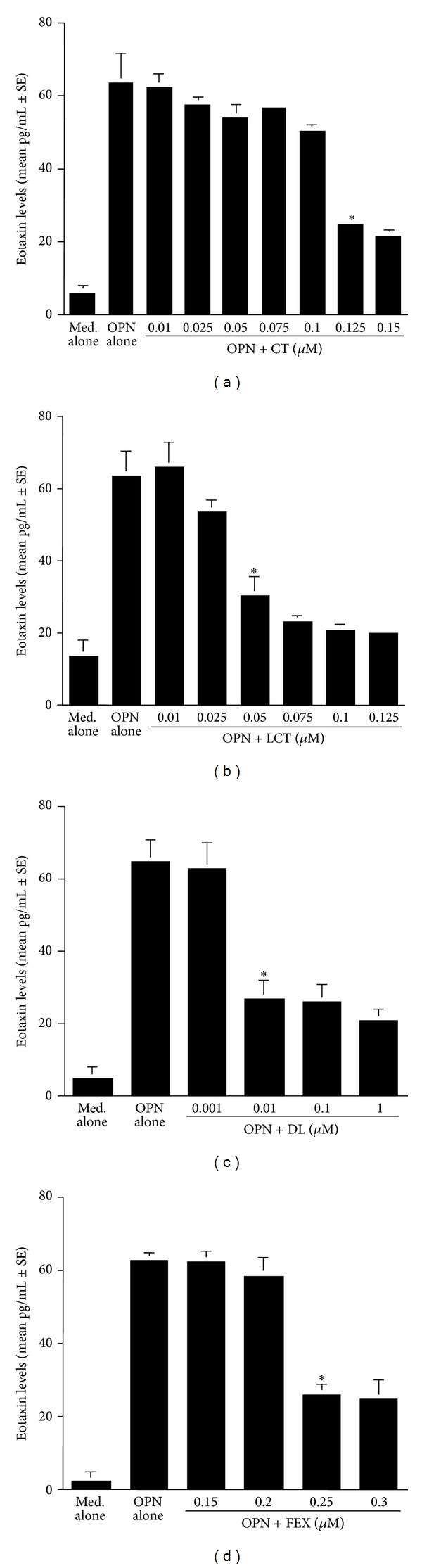
Influence of antihistamines on osteopontin (OPN)-induced Eotaxin production from nasal epithelial cells *in vitro*. Nasal epithelial cells obtained from five different subjects were stimulated with 250 ng/mL OPN in the presence of various concentrations of CT, LCT, DL and FEX for 24 h. Eotaxin levels in culture supernatants were examined by ELISA. *Significant (*P* < 0.05) versus OPN alone; (a) cetirizine (CT); (b) levocetirizine (LCT); (c) desloratadine (DL); (d) fexofenadine (FEX).

The second set of experiments was undertaken to examine the influence of antihistamines, CT, LCT, DL, and, FEX on the production of GM-CSF, RANTES, and Eotaxin from nasal epithelial cells after OPN stimulation. To do this, we first examined the optimal dose of OPN to produce factors from nasal epithelial cells. Epithelial cells were stimulated with various concentrations of OPN for 24 h and RANTES levels in culture supernatants were examined by ELISA. As shown in Figure 3, RANTES levels in culture supernatants were gradually increased by OPN stimulation, and the maximum levels were observed when OPN at more than 250 ng/mL was used for stimulation. We then examined the influence of antihistamines, CT, LCT, DL, and FEX, on GM-CSF production from epithelial cells in response to OPN stimulation. As shown in Figure 4(a), treatment of cells with CT at more than 0.1 *μ*M caused significant suppression of GM-CSF production, which was increased by the stimulation of OPN. Treatment of cells with LCT also caused suppression of GM-CSF production as in the case of CT, but the minimum concentration of the agent that caused significant suppression was 0.05 *μ*M (Figure 4(b)). Furthermore, the data in Figure 4 clearly showed that two other antihistamines, DL and FEX, could exert suppressive effects on GM-CSF production from nasal epithelial cells induced by OPN stimulation and that the minimum concentration of these two agents that caused significant suppression was 0.01 *μ*M for DL (Figure 4(c)) and 0.25 *μ*M for FEX (Figure 4(d)). Treatment of epithelial cells with antihistamines, CT, LCT, DL, and FEX, also caused the suppression of the production of both RANTES and Eotaxin at similar concentrations to those observed in the case of GM-CSF production, which were increased by OPN stimulation (Figures 5 and 6).

### 3.3. Influence of LCT on mRNA Expression for Factors Produced by Epithelial Cells

**Figure 7:**
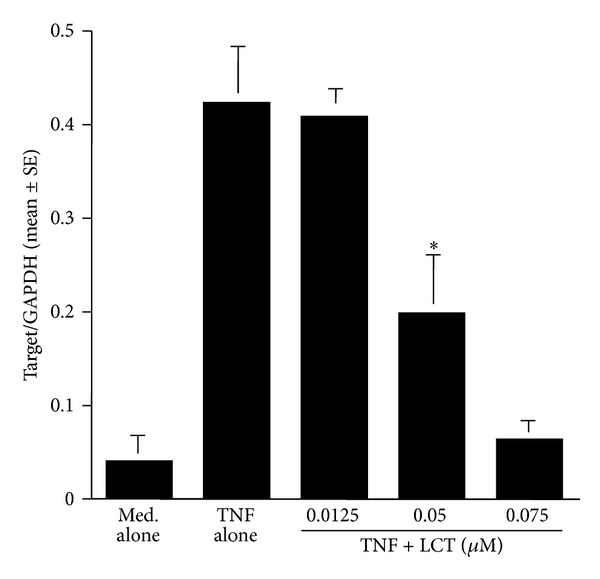
Influence of levocetirizine (LCT) on mRNA expression for osteopontin (OPN) in nasal epithelial cells* in vitro*. Nasal epithelial cells obtained from five different subjects were stimulated with 10 ng/mL TNF-*α* (TNF) in the presence of various concentrations of LCT for 12 h. mRNA expression was examined by real-time RT-PCR. *Significant (*P* < 0.05) versus TNF alone.

**Figure 8:**
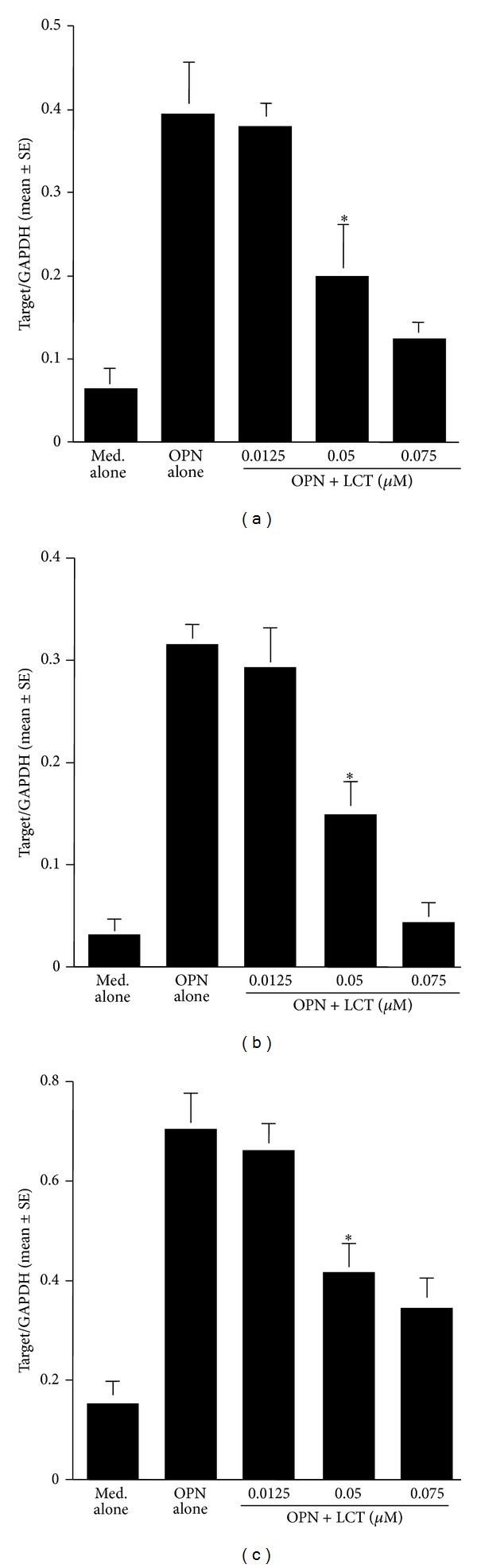
Influence of levocetirizine (LCT) on mRNA expression for GM-CSF, RANTES, and Eotaxin in nasal epithelial cells *in vitro*. Nasal epithelial cells were stimulated with 250 ng/mL osteopontin (OPN) in the presence of various concentrations of LCT for 12 h. mRNA expression was examined by real-time RT-PCR. *Significant (*P* < 0.05) versus OPN alone; (a) GM-CSF, (b) RANTES, and (c) Eotaxin.

The third set of experiments was undertaken to examine the influence of LCT on mRNA expression for OPN after TNF-*α* stimulation. Cells were cultured with 10.0 ng/mL TNF-*α* in the presence of the agents for 4 h. The levels of mRNA expression in cells were examined by real-time RT-PCR. As shown in Figure 7, treatment of epithelial cells with LCT at more than 0.05 *μ*M caused significant suppression of OPN mRNA expression, which was increased by TNF-*α* stimulation. In the second part of this set of experiments, we examined the influence of LCT on mRNA expression for GM-CSF, RANTES, and Eotaxin in epithelial cells after OPN stimulation. As shown in Figure 8, the addition of LCT at more than 0.05 *μ*M into cell cultures caused significant suppression of mRNA expression for GM-CSF, RANTES, and Eotaxin, which had been enhanced by OPN stimulation.

### 3.4. Influence of LCT on the Activation of Both NF-*κ*B and AP-1

**Figure 9:**
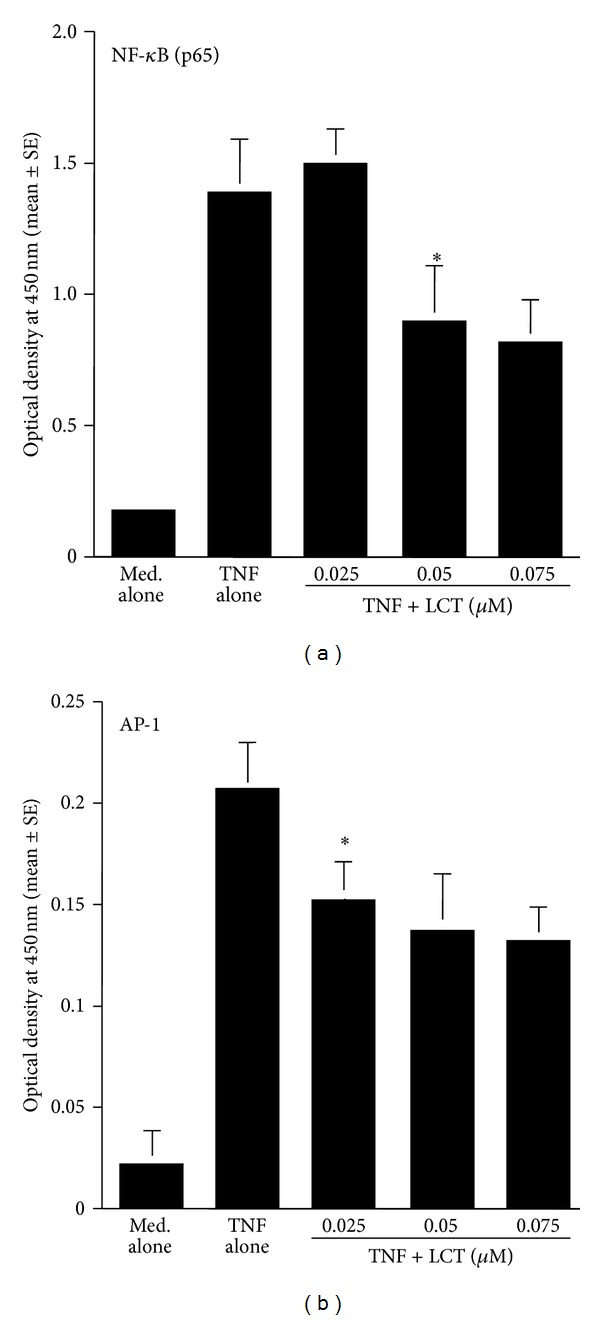
Influence of levocetirizine (LCT) on transcription factor activation in nasal epithelial cells *in vitro*. Nasal epithelial cells were stimulated with 10 ng/mL TNF-*α* (TNF) for 4 h. Transcription factor activation was examined by ELISA. *Significant (*P* < 0.05) versus TNF alone.

**Figure 10:**
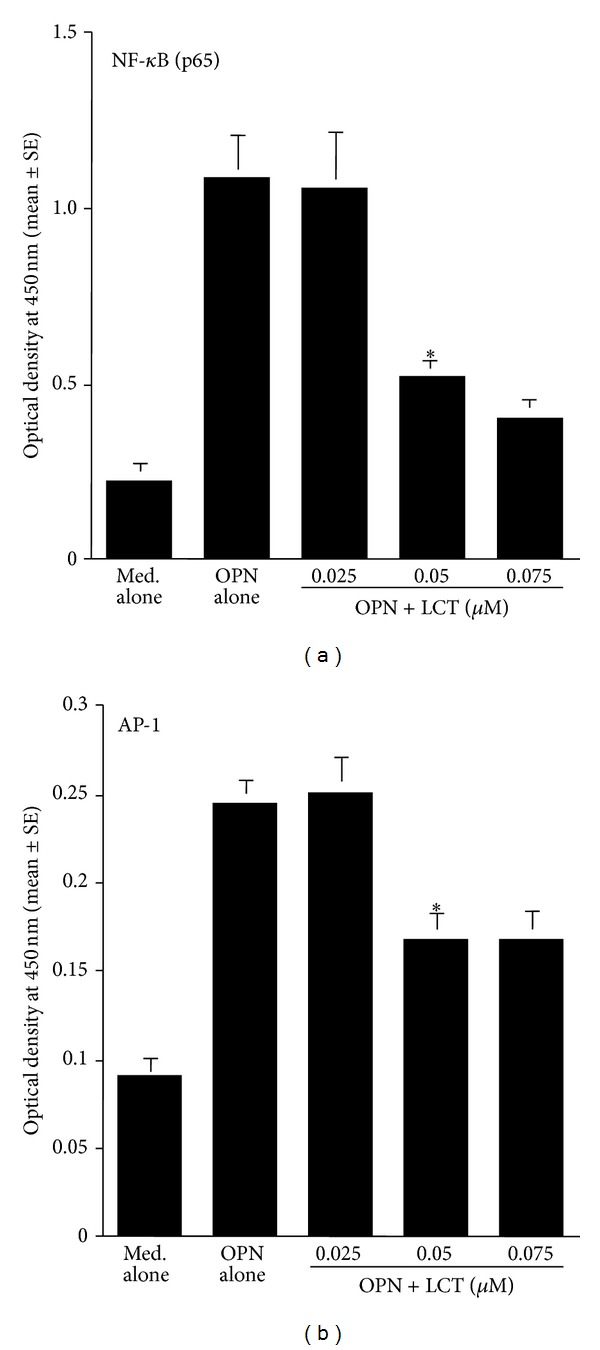
Influence of levocetirizine (LCT) on transcription factor activation in nasal epithelial cells* in vitro*. Nasal epithelial cells were stimulated with 250 ng/mL osteopontin (OPN) in the presence of various concentrations of LCT for 4 h. Transcription factor activation was examined by ELISA. *Significant (*P* < 0.05) versus OPN alone.

The fourth set of experiments was carried out to examine the influence of LCT on transcription factor activation in epithelial cells after the stimulation of either TNF-*α* or OPN. Epithelial cells were stimulated with 10.0 ng/mL TNF-*α* or 250 ng/mL OPN in the presence of the agent for 4 h. The nuclear extracts were prepared, and NF-*κ*B and AP-1 activities were measured by ELISA. As shown in Figure 9, treatment of cells with LCT at more than 0.05 *μ*M caused significant suppression of NF-*κ*B activation, which was increased by TNF-*α* stimulation. Furthermore, LCT could also suppress TNF-*α*-induced AP-1 activation, and the minimum concentration of the agent that caused significant suppression was 0.025 *μ*M, which is the half concentration that caused significant suppression of NF-*κ*B activation (Figure 9). We then examined the influence of LCT on the activation of both NF-*κ*B and AP-1 after OPN stimulation. As shown in Figure 10, treatment of cells with LCT at more than 0.05 *μ*M significantly suppressed the activation of both NF-*κ*B and AP-1 induced by OPN stimulation.

## 4. Discussion

OPN was originally discovered in bone as an extracellular matrix protein and was identified subsequently in many cell types in the immune system where it may be responsible for the pathogenesis of inflammatory responses and inflammatory diseases [1–6]. In human cases, it is reported that the levels of OPN in both sputum and bronchial lavage fluid (BALF) from asthmatic patients is much higher than that of healthy controls [8, 19]. Increased levels of OPN were also observed in IgE-mediated allergic diseases other than asthma, such as allergic conjunctivitis [20] and allergic rhinitis [21]. In regard to the influence of OPN on the effector cells in allergic immune responses, OPN could increase the chemotaxis of both mast cells and eosinophils *in vitro* [9, 22]. It is reported that administration of anti-OPN antibody significantly decreased the number of eosinophils in BALF obtained from an asthmatic mouse model [8] and that there is the significant correlation between OPN protein levels and the number of eosinophils in the sputum and BALF obtained from asthmatic patients [8, 19]. Airway remodeling is a prominent pathophysiological feature of allergic rhinitis [23] and asthma [22]. It is characterized by changes in structural abnormalities such as subepithelial fibrosis and mucus gland hypertrophy [22, 23]. In a murine model of allergen-induced airway remodeling, OPN expression is upregulated in lung tissues and BALF and is correlated with collagen content [24]. OPN is also reported to play an important role in angiogenesis, which is a characteristic feature of airway remodeling [19]. The present results clearly showed that CT and LCT could suppress the production of OPN from nasal epithelial cells in response to TNF-*α* stimulation. The minimum concentration of these agents that caused significant suppression of the production of these factors was 0.1 *μ*M for CT and 0.05 *μ*M for LCT, which is lower than therapeutic blood levels (approximately 0.348 *μ*M), when the agents at 5 mg were administered into humans [25]. The present data also clearly showed that DL at 0.01 *μ*M and FEX at 2.5 *μ*M, which are similar to therapeutic blood levels [25, 26], could exert the suppressive effects on OPN production from nasal epithelial cells after TNF-*α* stimulation. Taken together, it is strongly suggested that the suppressive effect of antihistamines, especially CT, LCT, DL, and FEX, on OPN production from nasal epithelial cells may be one of the therapeutic modes of action of these agents on allergic diseases such as asthma and allergic rhinitis.

Although there is evidence that OPN mediates cell-matrix interactions and cellular signaling through binding with integrin and CD44 receptors, which are responsible for inflammatory cell migration and tissue remodeling [27], several types of cytokines and chemokines are accepted to play roles in the development of pathophysiological changes in allergic diseases. Therefore, the second part of the experiments was undertaken to examine the influence of OPN on factor production from nasal epithelial cells and whether antihistamines, CT, LCT, DL, and FEX, could inhibit OPN-induced factor production. The present results clearly showed that antihistamines examined in this study could suppress the ability of epithelial cells to produce GM-CSF, RANTES, and Eotaxin, which were increased by OPN stimulation. Although the minimum concentration of CT and LCT that caused significant suppression was 0.1 *μ*M and 0.05 *μ*M, which are much lower than therapeutic blood levels (approximately 0.348 *μ*M) [25], the significant suppressive effects of DL and FEX were first observed when cells were treated with these agents at therapeutic blood concentrations. GM-CSF is a pleiotropic cytokine that can stimulate the maturation and function of hematopoietic cells such as neutrophils, macrophages, and eosinophils [28, 29]. GM-CSF can prolong eosinophil survival and enhance the ability of eosinophils to produce harmful molecules such as superoxide and arachidonic acid [29]. Eotaxin and RANTES induce chemotaxis as well as specifically activating eosinophils [17]. Several studies demonstrated the specificity of these chemokines for attraction and activation of eosinophils [30–32] and imply their participation in the recruitment of eosinophils to the site of allergic inflammation [30–32], suggesting that the suppressive activity of antihistamines on eosinophil chemoattractants and activators constitute the clinical efficacy of these agents on allergic diseases such as asthma and allergic rhinitis. This speculation may be supported, in part, by the observation that oral administration of either CT (10 mg daily) or LCT (5 mg daily) for 4 to 12 weeks in patients with allergic rhinitis could improve clinical symptoms with the inhibition of both eosinophil infiltration and activation in nasal mucosa [14, 15].

OPN engages a number of receptors such as integrins *α*_*v*_ and *β*1, and it is also a ligand for CD44 [22, 33]. These receptors directly or indirectly activate cellular signaling pathways such as p38 MAPK, NF-*κ*B, and AP-1, allowing OPN to mediate cell-matrix interaction and factor production [22, 33]. It is also reported that OPN production from airway cells requires the activation of transcription factors, NF-*κ*B and AP-1, after inflammatory stimulation [13]. Our previous work clearly showed that second-generation histamine H_1_ receptor antagonists, such as FEX, epinastine, and oxatomide, could suppress transcription factor activation induced by inflammatory and antigenic stimulation [34, 35], suggesting that antihistamines may suppress transcription factor activation, which is responsible for mRNA expression and results in the inhibition of factor production. This speculation may be supported by the present observations showing that LCT at more than 0.05 *μ*M could suppress mRNA expression for GM-CSF, RANTES, and Eotaxin through the suppression of NF-*κ*B and AP-1 activation.

## 5. Conclusion

This is the first report showing that antihistamines, especially CT, LCT, DL, and FEX, could suppress the OPN production and OPN-induced chemotactic factor, GM-CSF, RANTES, and Eotaxin, production from nasal epithelial cells at similar or lower than therapeutic blood levels, which may be responsible for the attenuating effect of these agents on allergic diseases such as asthma and allergic rhinitis.
